# Intraspecific Variability of Xylem Hydraulic Traits of *Calligonum mongolicum* Growing in the Desert of Northern Xinjiang, China

**DOI:** 10.3390/plants13213005

**Published:** 2024-10-28

**Authors:** Quanling Zhang, Hui Shen, Lan Peng, Ye Tao, Xiaobing Zhou, Benfeng Yin, Zhiqiang Fan, Jing Zhang

**Affiliations:** 1 Province Key Laboratory of the Biodiversity Study and Ecology Conservation in Southwest Anhui, College of Life Sciences, Anqing Normal University, Anqing 246133, China; zhangquanling0710@163.com; 2 State Key Laboratory of Desert and Oasis Ecology, Key Laboratory of Ecological Safety and Sustainable Development in Arid Lands, Xinjiang Institute of Ecology and Geography, Chinese Academy of Sciences, Urumqi 830011, China; shenhui20@mails.ucas.ac.cn (H.S.); penglan19@mails.ucas.ac.cn (L.P.); taoye@ms.xjb.ac.cn (Y.T.); zhouxb@ms.xjb.ac.cn (X.Z.); yinbf@ms.xjb.ac.cn (B.Y.); 3 Xinjiang Key Laboratory of Biodiversity Conservation and Application in Arid Lands, Xinjiang Institute of Ecology and Geography, Chinese Academy of Sciences, Urumqi 830011, China; 4 University of Chinese Academy of Sciences, Beijing 100049, China; 5 College of Resource and Environment Sciences, Xinjiang University, Urumqi 830017, China; 6 Xinjiang Field Scientific Observation Research Station of Tianshan Wild Fruit Forest Ecosystem, Yili Botanical Garden, Xinjiang Institute of Ecology and Geography, Chinese Academy of Sciences, Urumqi 830011, China

**Keywords:** desert shrub, xylem anatomical traits, intraspecific variability, environmental gradient, ecological adaptation strategy

## Abstract

Plant hydraulic traits are essential for understanding and predicting plant drought resistance. Investigations into the mechanisms of the xylem anatomical traits of desert shrubs in response to climate can help us to understand plant survival strategies in extreme environments. This study examined the xylem anatomical traits and related functional traits of the branches of seven *Calligonum mongolicum* populations along a precipitation gradient, to explore their adaptive responses to climatic factors. We found that (1) the vessel diameter (*D*), vessel diameter contributing to 95% of hydraulic conductivity (*D*_95_), hydraulic weighted vessel diameter (*Dh*), vessel density (*VD*), percentage of conductive area (*CA*), thickness-to-span ratio of vessels ((*t/b*)^2^), and theoretical hydraulic conductivity (*K_th_*) varied significantly across sites, while the vessel group index (*V_g_*), wood density (*WD*), and vulnerability index (*VI*) showed no significant differences. (2) Principal component analysis revealed that efficiency-related traits (*K_th_*, *D_h_*, *D*_95_) and safety-related traits (*VI*, *VD*, inter-wall thickness of the vessel (*t*)) were the primary factors driving trait variation. (3) Precipitation during the wettest month (PWM) had the strongest influence, positively correlating with (*t/b*)^2^ and negatively with *D*, *D*_95_, *D_h_*, *CA*, and *K_th_*. (4) Structural equation modeling confirmed PWM as the main driver of *K_th_*, with indirect effects through *CA*. These findings indicate that *C. mongolicum* displays high plasticity in xylem traits, enabling adaptation to changing environments, and providing insight into the hydraulic strategies of desert shrubs under climate change.

## 1. Introduction

Plant hydraulic traits are structural characteristics and physiological functions that have evolved to assist plants in adapting to the water conditions of their habitats. These traits reflect the essential aspects of water absorption, transport, utilization, and loss in plants [1], and they serve as a focal point for integrating plant and ecosystem functions [2]. As the global climate warms and dries, plants will face more frequent and severe droughts. The adaptability of plant hydraulic traits is vital for plant growth, physiology, and overall development. Understanding the plant–water relationship across environmental gradients is imperative to predict vegetation dynamics and species composition during future global changes [3].

The xylem is a specialized tissue for long-distance water transport in plants. It connects the roots to the upper parts of plants and is primarily composed of conductive elements, fibers, and parenchymal cells. Vessels (or tracheids) transport water and inorganic ions [4]. The Hagen–Poiseuille law states that the larger the diameter of xylem vessels, the higher the water transport efficiency [5], implying that anatomical characteristics significantly affect water transport efficiency in plants. Conduit diameters in the xylem of woody plants vary from <5 to >700 µm [6]. Plants in warm and humid environments tend to be taller, with larger vessel diameters and higher hydraulic conductivity [3,7], than those in arid regions, which tend to be smaller, with narrower vessel diameters that exhibit a stronger resistance to embolism [8,9]. Drought deciduous species exhibit wider vessels than evergreen species at the same plant height [10]. It can be clearly seen that the anatomical and structural characteristics of xylem vary greatly at the organ level (root, stem, and leaf) [6] and according to their functional type (liana, shrub, and tree) [11] and wood type (diffuse-porous and ring-porous) [12].

The xylem serves as a conduit for water transport and as a support structure. Previous studies have reported that the wood density (*WD*) and inter-wall thickness of the vessel (*t*) to lumen (*b*) ratio (*t/b)*^2^ are associated with the risk of vessel collapse under stress [13,14,15]. The higher the (*t/b*)^2^, the denser the wood, both of which can prevent xylem collapse under high tension, thereby increasing embolism resistance [13,14,16]. Species in arid regions have a higher (*t/b*)^2^ and greater *WD* than those in humid environments [17]. *WD* is becoming increasingly important as a plant functional trait, particularly in tree biomechanical support and hydraulic safety [18]. The plant economic spectrum indicates that fast-growing species have low wood density, while slow-growing species have high wood density. Higher *WD* is associated with smaller vessel diameters, thicker vessel walls, increased water transport safety, and reduced hydraulic efficiency [19]. However, the functional significance of wood density, particularly its role in sustaining tree growth under water stress, remains controversial. Liang et al. [20] and Greenwood et al. [21] found a positive relationship between *WD* and drought resistance based on a global synthesis of seedlings and measurements of adult trees. However, some regional studies, including that of Hoffmann et al. [22], reported that in temperate forests, species with high *WD* exhibit limited ability to regulate plant water potential, due to their insensitivity to water stress. Therefore, species with high *WD* exhibit higher resistance and higher mortality rates under extremely severe drought conditions.

Previous studies have reported that plant functional traits do not operate independently, but adapt to changing external environments by coordinating multiple trait adjustments [9,23]. Baas et al. [24] proposed the “trade-off triangle” hypothesis, which implies a trade-off between xylem water transport efficiency, safety, and mechanical strength. However, excessive xylem construction may eliminate this trade-off [25]. López et al. [26] reported a trade-off between *WD* and hydraulic efficiency in *Hakea leucoptera*, but not in *Hakea dactyloides*. Additionally, Zhang et al. [27] investigated 12 wood traits of 60 angiosperm tree species from temperate, Mediterranean, and tropical climates and reported a negative correlation between *WD* and theoretical hydraulic conductivity (*K_th_*). However, in the arid regions of Northwest China, the *WD* of the desert shrub *Reaumuria soongarica* is unrelated to water transport efficiency (*K_th_*) [28]. These studies demonstrate that the trade-off between mechanical traits and hydraulic efficiency varies with species and study region.

Plasticity refers to variations in plant traits caused by external environmental changes. Plant hydraulic traits are susceptible to environmental changes, according to studies conducted at the local and regional scales. Previous studies have investigated climatic effects on xylem anatomical and hydraulic traits [3,29,30,31]. For example, simulated rainfall experiments revealed that xylem anatomical adaptation to rainfall changes differs among typical shrubs on the Loess Plateau. Increased water treatment significantly increased the vessel diameter of *Salix psammophila*, but exhibited no significant impact on the xylem structure of *Caragana korshinskii* [32]. Studies on 316 angiosperms from Southwest China reported that the mean vessel diameter (*D*) and potential hydraulic conductivity (*K_th_*) were positively correlated with the mean annual temperature (MAT) and mean annual precipitation (MAP) [33,34]. However, a study on ten *Caragana* shrub species across a climate gradient reported a negative correlation between leaf hydraulic conductivity and MAP [31]. Furthermore, Schuldt et al. [30]. reported that in the widely distributed species *Fagus sylvatica*, the diameter of the stem xylem vessel did not correlate with MAP, despite significant variation across different sites. Further studies reported that reduced summer precipitation resulted in a significant decrease in *D*, indicating that seasonal climate changes significantly affect xylem hydraulic traits [3,29]. Simultaneously, global studies confirm that growing season temperatures and precipitation significantly affect hydraulic conductivity [3]. Besides this, hydraulic traits vary with atmospheric humidity [9], vapor pressure deficit [35], and soil fertility [36].

The effect of climatic factors on xylem hydraulic traits at the interspecific level has been extensively studied, which is crucial for understanding species distribution and predicting plant responses to climate change [23,37]. However, intraspecific variation in hydraulic traits has been less studied, despite its significance for species adaptation to environmental conditions [20,30]. Previous studies have reported that intraspecific trait plasticity may be a key factor in the ability of species to colonize new environments or resist environmental changes [38]. Transect studies provide a unique research approach, acting as natural laboratories for investigating the relationship between plant traits and climate change. Previous transect studies on hydraulic traits have primarily focused on tropical and subtropical regions, with significant research conducted in Eastern and Southwestern China [20,30,39], providing valuable insights into the spatial patterns of plant hydraulic traits. However, the primary driving factors behind spatial pattern changes in intraspecific hydraulic traits in arid regions are unclear and require further study.

The desert region of Northwest China is a typical ecologically fragile area due to global changes [40]. Xerophytic and hyper-xerophytic shrubs, semi-shrubs, and small trees comprise most vegetation in this region. *Calligonum mongolicum*, a perennial shrub or semi-shrub in the *Polygonaceae* family, is abundant and widely distributed in the desert region of China. It contributes significantly to wind prevention, sand fixation, and desert ecosystem stability [41]. Currently, anatomical structure studies primarily focus on leaves (assimilated branches) [42,43,44]. This study frequently employs indoor pot experiments and field-controlled experiments. However, studies on the spatial variation in xylem anatomical traits and their driving forces in this widespread desert species are rare. Here, we examined the anatomical structure of stem xylem vessels, mechanical strength, and hydraulic traits (theoretical hydraulic conductivity and vulnerability index) of seven *C. mongolicum* populations across environmental gradients in the desert region of Northern Xinjiang, China, and analyzed their relationship with climatic factors. We tested the hypothesis that (1) vessel diameter and hydraulic efficiency increase with precipitation and (2) there is a trade-off between “efficiency and safety” and “efficiency and mechanical strength” in hydraulic traits. This study aims to explore the changes in the xylem anatomical traits of *C. mongolicum* across different environmental gradients, to better understand its physiological and ecological adaptability, and to provide hydraulic evidence for predicting dynamic changes and stability in Northwestern desert vegetation communities in China under climate change.

## 2. Materials and Methods

### 2.1. Study Area

**Figure 1:**
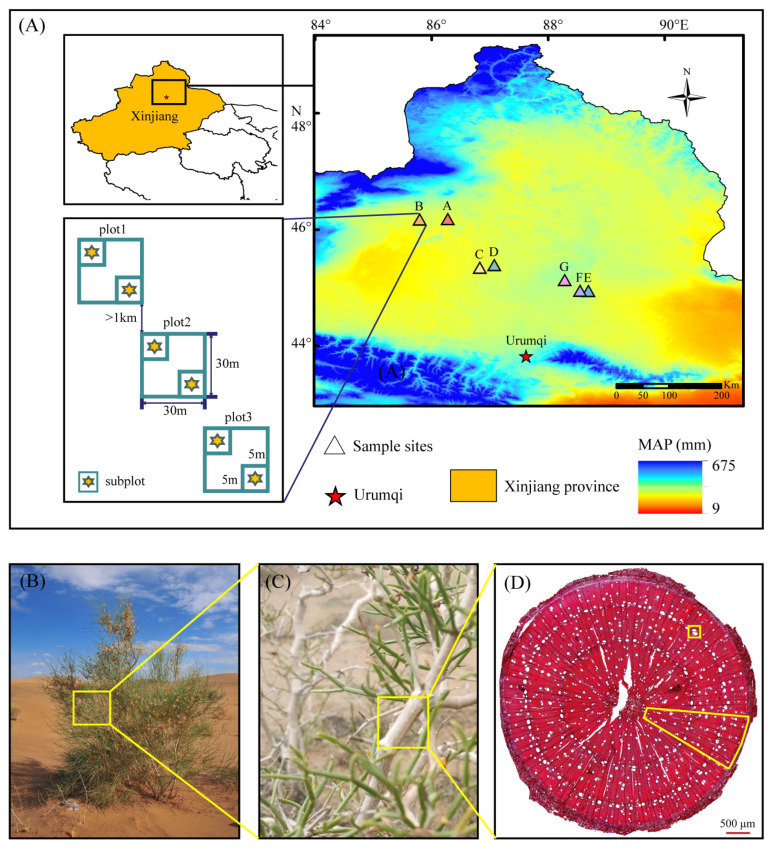
Sampling maps and measurements of the *C. mongolicum*. Notes: (**A**) Different sample sites (triangular sites) with different colors are sorted from low to high by MAP and marked A–G. The inset map displays the location, shape, and size of the sampling plots. (**B**) Shows the habitat and general morphology of the sampled individuals. Yellow rectangles indicate the specific sections where sampling occurred on the plant stems. (**C**) A close-up view of the collected branch samples. (**D**) Xylem cross-section images of *C. mongolicum*. The yellow sector is the measurement area. Yellow rectangle, a group of vessels.

The study area is in the northern desert region of Xinjiang province (Western China). Seven sample sites were selected along the sample transect from west to east where *C. mongolicum* is distributed. The study area includes Karama, Changji, Altay, and Tarbagatay (44.94–46.18° N, 85.79–88.69° E) (Table 1). The mean annual precipitation (MAP) and mean annual temperature (MAT) of the study area were 144.69–186.17 mm and 6.1–8.5 °C, respectively (Worldclim database: https://worldclim.org, accessed on 14 March 2023), and the aridity index (AI) was 0.09–0.12 (CGIAR-CSI: https://www.cgiar-csi.org, accessed on 27 March 2023). The sample sites were labeled A to G based on their MAP, ordered from small to large (Figure 1). The vegetation types in this study area were primarily shrubs, semi-shrubs, and small trees. In addition to *C. mongolicum*, the dominant shrubs and semi-shrubs included *Haloxylon ammodendron*, *Haloxylon persicum*, *Artemisia ordosica*, and *Ephedra distachya*.

### 2.2. Sample Plot Setting and Sample Collection

Here, *C. mongolicum* in the transect was selected as the research object, and field sampling was conducted in July 2022. Each sample site consisted of three 30 × 30 m plots, and the distance between the plots was >1 km. Two 5 × 5 m subplots were set on the diagonal of each plot. Ultimately, there were six subplots for each sample site. One individual was collected at each subplot, and at each sample site we selected 5–6 mature and healthy individuals of similar crown width and height. Mature, south-facing, well-grown branches with similar branch diameters were collected at the mid-height of the individual. After collecting, they were loaded in a collection bag and transported back to the laboratory for testing.

### 2.3. Measurement Methods

#### 2.3.1. Anatomical Structure of Xylem

The middle part of the branch, with a diameter of 4–5 mm or so, was selected to make paraffin sections. Branches were cut into 0.5 cm samples and fixed in FAA fixative for 24 h. Then, it was softened, dehydrated with ethanol, and made transparent with xylene. The samples were then placed in paraffin wax and cut into slices of 10–15 μm thickness using a Pfm rotary 3004 M microtome (pfm medical ag, Köln, Germany), stained with 1% safranin solution, and sealed with neutral balsam to create permanent slides. Finally, the samples were observed and photographed with the same magnification (×10, ×20) under an Olympus BX41 optical microscope (Olympus, Tokyo, Japan) after sealing on the slides with neutral balsam. These images were then combined to produce an integrated image covering the whole cross-section, using the PTGui panorama stitching tool (http://www.ptgui.com, accessed on 3 May 2023). Three complete sectors (30° each) were randomly selected from the cross-sectional images of each sample, and for subsequent calculations, each sector was analyzed in its entirety, excluding the pith and bark [20,28,45]. Table 2 illustrates all the traits measured in this study.

The Image-J software (ImageJ 1.45) “particle analysis function” was used to directly derive the mean vessel area (*Ā*, μm^2^) and the percentage of the conductive area (*CA*, %, which represents the proportion of total conduit area per unit area). The following traits were derived as follows: (1) The cross-section of the vessel was considered to be approximately circular. The *D* (μm) was calculated using the relationship between the circular area and the diameter, *D* = $\sqrt{4Ā/\pi }$. The hydraulic weighted vessel diameter (*D_h_*, μm), *D_h_* = $\frac{\sum D^{5}}{\sum D^{4}}$. The vessel diameter contributing 95% hydraulic conductivity (*D*_95_, μm) was determined by ranking all diameters raised to the fourth power in descending order (*D^4^*) and summing them (*D^4^*s) until their total was equal to 95% of the total conductance; vessel density (*VD*, no·mm^−2^), the number of vessels per unit area; the vessel grouping index (*V_g_*), the average number of vessels with continuous cell walls. (2) The thickness-to-span ratio (*t/b*)^2^ represented the ability of the vessel to resist embolization. The greater the value, the stronger the anti-embolism ability of the vessels [13,15]. A selected diameter in the *D_h_* ± 5 µm range of vessel pairs was used for measurements, *t* is the cell wall thickness between the two connected vessels, *b* is the average value of the long and short axes of the two vessels; the *WD* (*WD*, g·cm^−3^) was measured using the drainage method and is the ratio of the drying weight of the wood (at 70 °C for 48 h to a constant weight) to wood volume. (3) The theoretical hydraulic conductivity (*K_th_*, kg·m^−1^·MPa^−1^·s^−1^) reflects the water transport efficiency in the xylem and was calculated using Hagen–Poisetuille’s law [46]:

$$K_{\mathrm{th}}=\frac{D_{h}^{4}\pi \rho }{128\times \eta }\times VD$$

where *ρ* is the density of water at 20 °C (998.2 kg·m^−3^); *η* is the water viscosity at 20 ℃ (1.002 × 10^−9^ MPa·s).

Carlquist’s vulnerability index (*VI*, mm·m^−2^), which reflects the resistance of plants to drought-induced cavitation embolism [5,47], was calculated as follows:

$$\mathrm{VI}=\frac{D_{h}/1000}{VD/1000000}$$

#### 2.3.2. Climate Factor Acquisition

The global positioning system (Magellan GPS315, Magellan) was utilized to ascertain the latitude, longitude, and elevation of the plants. Twenty climate factors were extracted from the Worldclim (http://www.worldclim.org, accessed on 14 March 2023) and CGIAR-CSI (https://www.cgiar-csi.org, accessed on 27 March 2023) databases. WorldClim and CGIAR-CSI climate variables were all based on average values from 1970 to 2000 using high-resolution (30 arc-seconds) weather-station data. Collinearity between climate factors was removed through correlation analysis (Figure S1). MAT, MAP, and the aridity index were retained. The remaining climate factors were selected from the correlation coefficient |r| ≥ 0.9 to be retained. Eight climatic factors, including MAT, MAP, AI, the mean temperature of the driest quarter (TDQ), the mean temperature of the coldest quarter (TCQ), the precipitation of the wettest month (PWM), the precipitation of the driest month (PDM), and the precipitation of the wettest quarter (PWQ), were retained.

### 2.4. Data Analysis

Before statistical analysis, the data were tested for normality and variance homogeneity. If the data did not conform to the normal distribution or variance homogeneity, logarithmic transformation was used to improve the data normality. The quartile coefficient of dispersion (QCD) was used to assess trait variation, calculated for each trait as the ratio between half the interquartile range ((Q3 − Q1)/2) and the average of the quartiles ((Q1 + Q3)/2). QCD is less sensitive to outliers and skewed distributions than the coefficient of variation, making it suitable for a wider range of data types [23]. One-way analysis of variance (ANOVA) was used to compare xylem structure across different sites, followed by multiple comparisons using the least significant difference (*p* = 0.05). Pearson’s analysis was used to calculate the correlation between the two traits. The “Corrplot” package was used to analyze the correlations between traits and climatic factors. PCA was used to visualize the overall coordination and trade-offs among xylem traits. The “piecewiseSEM” package was used to construct structural equation modeling to clarify the direct and indirect influence of climatic factors on the xylem hydraulic traits of the stem of *C. mongolicum*. Data in the graphs are presented as mean ± standard error. All statistical analyses and mapping were performed using R software (version 4.3.0).

## 3. Results

### 3.1. Intraspecific Variation of Hydraulic Traits in Xylem

**Figure 2:**
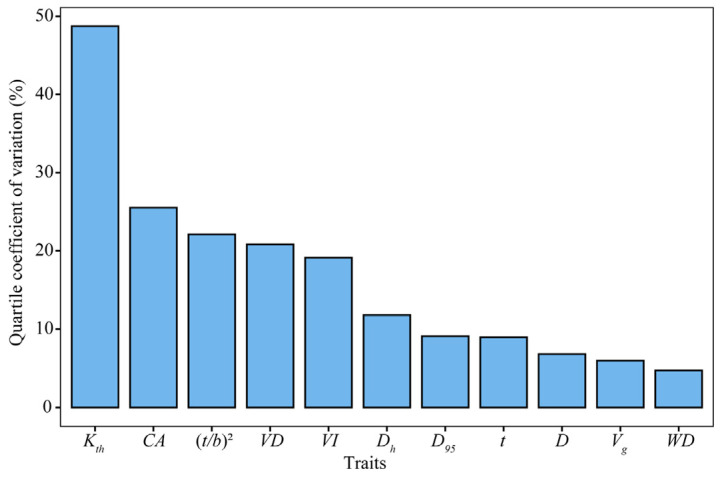
Quartile coefficient of dispersion of the stem xylem hydraulic traits of *C. mongolicum*.

Hydraulic traits in the stem xylem of *C. mongolicum* exhibited different degrees of variability at the intraspecific level. Of the 11 measured hydraulic traits, Kth exhibited the highest variability of 48.73%, followed by *CA* and (*t/b*)^2^ with 25.52% and 22.1%. *WD* exhibited the lowest variability of 4.71%, followed by *V_g_* and *D* with 5.97% and 6.79% (Figure 2).

**Figure 3:**
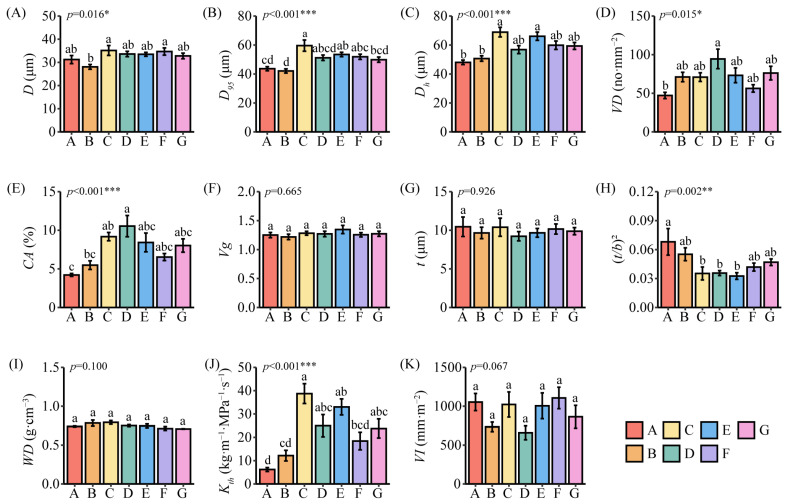
Analysis of differences in stem xylem hydraulic traits of *C. mongolicum* among sample sites. Notes: (**A**) Mean vessel diameter; (**B**) hydraulic weighted vessel diameter; (**C**) vessel diameter contributing 95% hydraulic conductivity; (**D**) vessel density; (**E**) percentage of conductive area; (**F**) vessel grouping index; (**G**) inter-wall thickness of the vessel; (**H**) thickness-to-span ratio of vessels; (**I**) wood density; (**J**) theoretical hydraulic conductivity; (**K**) Carlquist’s vulnerability index. Error bars are standard errors. Different letters indicate significant differences at *p* < 0.05. Different sample sites with different colors are sorted from small to large by MAP and marked A–G. * *p* < 0.05, ** *p* <  0.01, *** *p* < 0.001.

One-way ANOVA and the least significant difference were performed for 11 stem xylem hydraulic traits of *C. mongolicum* at seven sample sites (Figure 3). One-way ANOVA revealed that *D*, *D*_95_, *D_h_*, *VD*, *CA*, (*t/b*)^2^, and *K_th_* were significantly different (*p* < 0.05) across sample sites (Figure 3A–E,J), whereas *V_g_*, *t*, *WD*, and *VI* (Figure 3F–I) were non-significantly different. The least significant difference revealed the extent of differences in xylem structural traits among various sample sites. As for anatomical structure traits, *D* and *D*_95_ were the highest at Site C and the lowest at Site B; *D* and *D*_95_ were significantly lower at Site B than at Site C, with non-significant differences observed among the remaining sample sites (Figure 3A,B). *D_h_* was the highest at Site C and the lowest at Site A. Non-significant differences were observed among the remaining sample sites, except for Sites A and B, which were significantly lower than Sites C and E (Figure 3C). *VD* and *CA* were the highest at Site D and the lowest at Site A; *VD* was significantly lower at Site A than at Site D, and *CA* was significantly lower at Site A and B than at Site D. Non-significant differences were observed among the remaining sample sites (Figure 3D,E). As for mechanical strength traits, (*t/b*)^2^ was the highest at Site A and the lowest at Site E; (*t/b*)^2^ was significantly higher at Site A than at Sites C, D, and E, and non-significant differences were observed among the rest of the sample sites (Figure 3H). As for hydraulic functional traits, *K_th_* was the largest at Site C and the lowest at Site A; *K_th_* was significantly lower at Site A than at Sites C, D, E, and G, and significantly higher at Site C than at Sites A, B, and F (Figure 3J).

### 3.2. Correlation Analysis of Stem Xylem Hydraulic Traits

**Figure 4:**
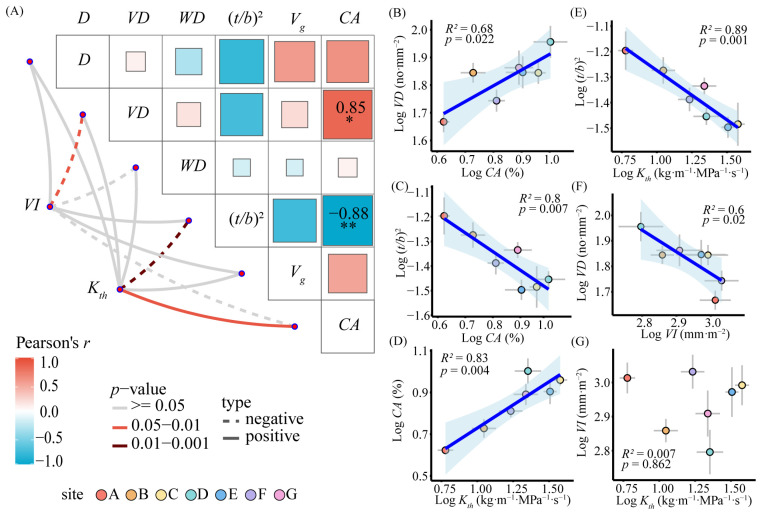
Correlations between the stem xylem hydraulic traits. Notes: Pearson’s correlation analysis (**A**) and general linear regression analysis (**B**–**G**) of hydraulic functional traits and anatomical traits of *C. mongolicum*. (**A**) The triangular section in the upper left corner depicts the relationship between the traits, where the color gradient indicates Pearson’s correlation coefficient. The solid line and dashed lines represent positive and negative correlations, respectively. The line color indicates statistical significance; red is an extremely significant correlation, dark red is a significant correlation, and gray is a non-significant correlation. (**B**–**G**) The points with different colors in the linear regression analysis represent different sample sites. The blue lines represent the fitted curves or relationship lines obtained through general linear regression analysis, which are statistically significant (*p* < 0.05), while shaded areas represent the 95% confidence interval. The asterisk indicates significant correlations. * *p* <  0.05; ** *p* <  0.01.

Correlation analysis of the xylem structure traits of *C. mongolicum* revealed that *CA* was significantly positively correlated with *VD* (*p* < 0.05, Figure 4A,B) and significantly negatively correlated with (*t/b*)^2^ (*p* < 0.01, Figure 4A,C). *D*, *WD*, and *V_g_* were non-significantly correlated with all traits. The hydraulic efficiency indicator *K_th_* demonstrated a significant positive correlation with *CA* (*p* < 0.05, Figure 4A,D) and a significant negative correlation with (*t/b*)^2^ (*p* < 0.01, Figure 4A,E). The hydraulic safety indicator *VI* demonstrated a significant negative correlation with *VD* (*p* < 0.05, Figure 4A,F). The hydraulic efficiency indicator *K_th_* was non-significantly correlated with the hydraulic safety indicator *VI* (*p* > 0.05, Figure 4G).

### 3.3. Principal Component Analysis of Xylem Hydraulic Traits

**Figure 5:**
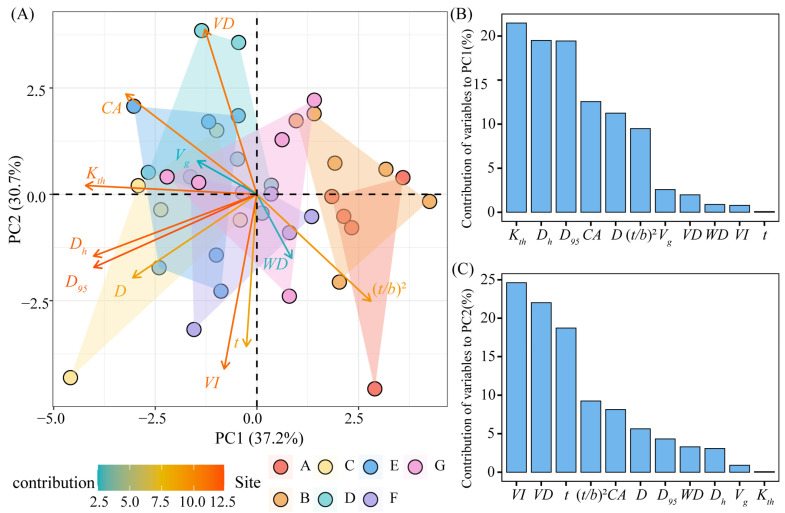
PCA of stem xylem hydraulic traits of *C. mongolicum*. Notes: (**A**) Principal component analysis of stem xylem hydraulic traits of *C. mongolicum*; (**B**) the contribution of each trait to PC1; (**C**) the contribution of each trait to PC2. The points of different colors represent the different sample sites.

Figure 5 depicts the results of the principal component analysis of the xylem hydraulic traits of *C. mongolicum*. The first two principal components (PC1 and PC2) accounted for 67.9% of the total variation for all xylem hydraulic traits, with explained variances of 37.2% and 30.7%, respectively (Figure 5A). The highest contributing hydraulic traits in the first PC axis were *K_th_*, *D_h_*, and *D*_95_ (Figure 5B), while the highest hydraulic traits in the second PC axis were *VI*, *VD*, and *t* (Figure 5C).

### 3.4. Effects of Climatic Factors on Changes in Hydraulic Traits

**Figure 6:**
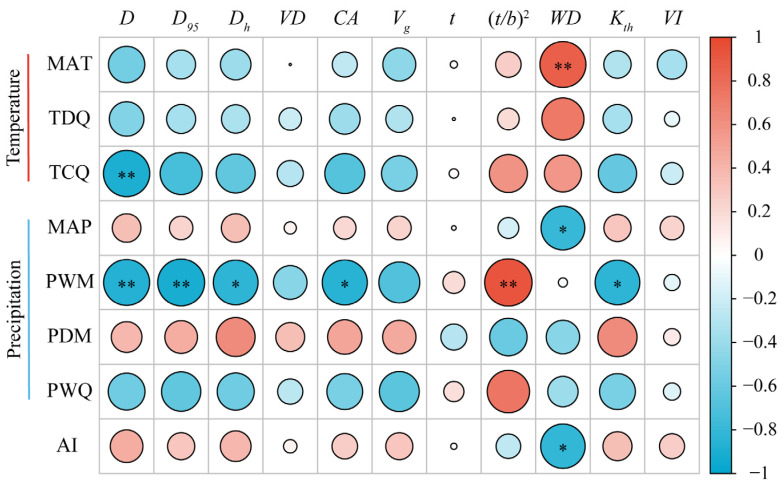
Relationships between stem xylem hydraulic traits and climatic factors. Notes: The heatmap displays a significant correlation (calculated by Pearson’s correlation analysis). The color of the circle indicates a positive correlation (red) or negative correlation (blue), while color intensity signifies the strength of the correlation. * *p* < 0.05; ** *p* < 0.01.

Figure 6 depicts the results of the correlation analysis between eight climatic factors and 11 stem xylem hydraulic traits of *C. mongolicum*. *D* exhibited a significant negative correlation with TCQ and PWM; *D*_95_, *D_h_*, *CA*, and *K_th_* exhibited significant negative correlations with PWM; (*t/b*)^2^ displayed a significant positive correlation with PWM; *WD* displayed a significant positive correlation with MAT and was significantly negatively correlated with AI and MAP; *VD*, *V_g_*, *t*, and *VI* were all non-significantly correlated with climatic factors.

**Figure 7:**
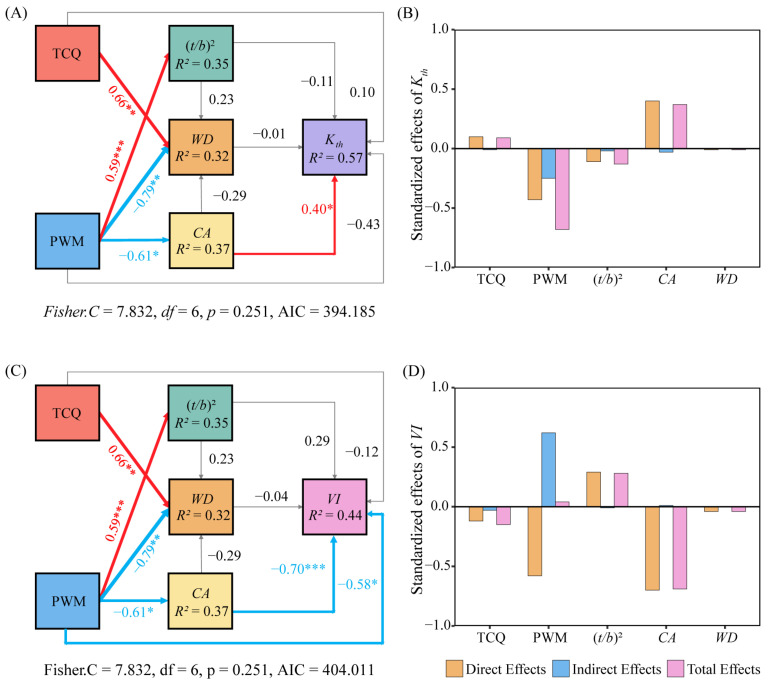
Driving factors of stem xylem hydraulic traits of *C. mongolicum*. Notes: (**A**,**C**) Structural equation modeling illustrating the impact of climatic factors on *K_th_* and *VI*. (**B**,**D**) The histogram depicts the standardized impacts of driving factors. The red and blue arrows represent statistically significant positive and negative effects (*p*  <  0.05), while the gray arrows indicate a non-significant relationship. The numerical value next to the arrow represents the standardized path coefficient. The width of the arrow is directly proportional to the strength of the path coefficient. R^2^ donates the proportion of variance that is accounted for by the model. Significant levels of each predictor are * *p*  <  0.05; ** *p* < 0.01; *** *p* < 0.001.

Structural equation modeling (SEM) was used to further analyze the pathways and mechanisms in which climatic factors influence the stem xylem hydraulic traits of *C. mongolicum*. SEM accounted for 57% and 44% of *K_th_* and *VI*, respectively (Figure 7A,C). Climate factors have a direct or indirect impact on hydraulic functional traits. The effect of PWM on *K_th_* was observed through its impact on *CA*, resulting in a total effect of –0.68 (Figure 7B). PWM exhibited a significant and direct negative impact on *VI* (direct effect –0.58). Additionally, PWM indirectly affected *VI* by affecting *CA*, resulting in a total effect of 0.04 (Figure 7C,D). Although TCQ exhibited a non-significant effect on hydraulic functional traits, it displayed the most significant total effect on *VI* at –0.15 (Figure 7D).

## 4. Discussion

### 4.1. Characteristics of Spatial Variation in Hydraulic Traits

Plastic variation in plant functional traits is crucial for adapting to environmental changes [48]. The conservative strategy increases stress resistance by minimizing trait plasticity and maximizing the use of limited resources. However, the acquisition strategy, characterized by higher phenotypic plasticity, enables rapid responses and the efficient utilization of abundant resources [49]. This study demonstrated significant differences in the variability of each trait across the same environmental gradient. *K_th_* exhibited the most significant variation, while *WD* displayed the least variability, with tenfold differences in the quartile dispersion coefficient. This was consistent with the research findings of Rosas et al. [23] on six dominant tree species in Northeastern Spain (three Fagaceae and three Pinaceae). Specifically, when comparing exact measurements in this study, we observed that sapwood-specific hydraulic conductivity (*K_s_*) exhibited the highest degree of variation, while *WD* displayed the lowest. The primary cause of variation was interspecific. However, another previous study on the dominant shrub *R. soongarica* in the desert region of Northwestern China reported that (*t/b*)^2^ exhibited the highest degree of variability, followed by *K_th_* [28].

Previous studies have reported that the hydraulic conductivity of plants has high plasticity, which improves the ability of plants to adapt to environmental conditions [50,51]. The high variability of *K_th_* can be attributed to fluctuations in environmental conditions and adaptations in plant morphology and intraspecific anatomy [23,38]. According to the Hagen–Poiseuille law, small variations in vessel diameter can cause significant alterations in hydraulic efficiency. Although *D* was not significant between sample sites (except sites B and C), it can still coordinate with other structural traits to adjust for changes in *K_th_* [9]. Conversely, we found that the hydraulic traits *VI* and *t*, indicative of security, were non-significant between the different sample sites, consistent with the findings of Shen et al. [28]. In arid habitats, plants will often be exposed to drought stress; therefore, it is imperative to ensure their hydraulic safety. Pritzkow et al. [52] reported that traits related to hydraulic safety were under strong genetic control using transplantation experiments, and Sanchez-Martinez et al. [53] reported that xylem embolism resistance (*P_50_*) is highly conserved on an evolutionary scale in 2027 species worldwide. Furthermore, plants require conserved traits to improve their resistance to environmental stresses [54]. Consequently, we hypothesized that *VI* and *t* are phylogenetically limited to some extent, ensuring that the differences between different sites are non-significant.

*WD*, the trait with the lowest variability, reflects the carbon investment of plants in its water-conducting tissues relative to the stem and is associated with the life-history strategy, functional physiology, mechanical properties, and structure of plants [18]. Various cellular components of the xylem (vessels, fiber cells, parenchyma cells) collectively determine the size of the *WD*. These components undergo coordinated changes in response to varying climate conditions; however, they do not necessarily change functional trade-offs [27]. Additionally, previous studies have reported that *WD* has phylogenetically conserved characteristics, which renders it less susceptible to environmental influences [55,56].

### 4.2. Coordination and Trade-Offs in Hydraulic Traits

The xylem’s structure adapts to environmental changes via coordinated adjustments among various traits rather than responding independently [9]. Our study demonstrated a significant positive correlation between *CA* and *VD*, indicating that increasing *VD* leads to higher *CA*. This same pattern was found by García-Cervigón et al. [57] in *Nothofagus pumilio* stem anatomy. Mechanical support of the xylem is closely associated with water transport [58,59]. The (*t/b*)^2^ represents the ability of xylem vessels to resist implosion under negative pressure. If the (*t/b*)^2^ is too small, it increases the risk of tube collapse and lowers safety [14]. Here, *K_th_* was significantly positively correlated with *CA* and significantly negatively correlated with (*t/b*)^2^, while *CA* was significantly negatively correlated with (*t/b*)^2^, demonstrating the trade-off between mechanical support and the hydraulic efficiency of *C. mongolicum* [60]. We hypothesized that decreasing the xylem (*t/b*)^2^ of *C. mongolicum* would increase *CA* and improve water transport efficiency. Additionally, *VI* was only significantly negatively correlated with *VD* in this study, implying that the higher the *VD*, the lower the embolism susceptibility. Islam et al. [35] reported that an increase in *VD* results in the appearance of more small vessels, which in turn allow the xylem to maintain lower hydraulic pressures and improves its embolism resistance, thereby avoiding drought-induced cavitation.

The trade-off between xylem water transport efficiency and safety is highly controversial, and varies depending on the subject, organ level, study area, and study scale [27,29,31,61]. We found a non-significant trade-off between *K_th_* and *VI* in this study, consistent with the findings of Maherali et al. [62], which demonstrated a safety–efficiency trade-off in evergreen angiosperms and conifers in deciduous angiosperms. PCA revealed a strategy for hydraulic efficiency and safety in *C. mongolicum*, with the first trait axis associated with water transport efficiency and the second with hydraulic safety. All sample sites were primarily separated along the first axis, whereas the second axis was less discriminatory, implying that xylem water transport efficiency was the main factor distinguishing different *C. mongolicum* sample sites, which could be seen in the results of variance analysis and tests of difference. A previous study on three broadleaf species in Central Europe reported that the xylem safety-related wood *P_50_* and leaf turgor loss point (*P_TLP_*) traits are highly conserved [38]. However, previous studies have reported that xylem hydraulic conductivity is susceptible to the environmental climate [20,30,35,37].

The intraspecific variation in plants determines the suitable range and survival persistence of the species [54], and larger *K_th_* variability reflects the water acquisition strategy of *C. mongolicum* in arid regions. Under drought stress, osmotic solutes (soluble sugars, proline) and antioxidant enzymes were significantly upregulated in *C. mongolicum*, contributing to maximizing water uptake and reducing oxidative damage [63]. Additionally, assimilated branches of *C. mongolicum* exhibited higher photosynthetic rate and water use efficiency than species with leaves [42]. Experiments have confirmed that assimilated branches of *C. mongolicum* have a leaf-absorbing ability to supplement stem water with water absorbed from the atmosphere [64]. Although this study did not observe a direct functional trade-off between the efficiency and safety of the hydraulic system of *C. mongolicum* at the tissue level, the embolism resistance of the xylem depends on the structure of pits between vessels, including the pit membrane thickness, pit membrane diameter, and pit opening ratio [14]. We have found that *C. mongolicum* has special vestured pits (unpublished data), a structure commonly found in desert species, which can enhance xylem safety [65]. Therefore, a comprehensive analysis is required to investigate the xylem hydraulic trade-off relationship with the xylem vessel structure and other xylem tissue structures. Connectivity (functional connection) between tissues will affect the hydraulic efficiency and safety of the xylem.

### 4.3. The Driving Effects of Climatic Factors on Hydraulic Traits

Previous studies have investigated trait–environment relationships from within species to communities to predict vegetation dynamics during climate change [3,30,33,66]. Precipitation is the primary source of water at a regional scale, and fluctuations in precipitation can influence the hydraulic traits of trees [67]. It is generally observed that *D* and *K_th_* exhibit a positive correlation with MAP [33,34]. For instance, *D_h_* and *K_th_* increase with MAP in *Castanopsis fargesii* populations in humid areas of southern China [20]. In our study, while an increase in MAP showed a weak positive correlation with *D* and *K_th_*, the relationship did not reach statistical significance. Similarly, no significant correlation between *D* and *K_th_* with MAP was found in a study of *Reaumuria soongarica* populations in desert areas [28], consistent with our findings. A study on the widespread species *Cordia alliodora* found that sapwood-specific hydraulic conductivity (*K_s_*) and leaf-specific hydraulic conductivity (*K_l_*) did not significantly change with increasing MAP. This suggests that *C. alliodora* maintains stable hydraulic efficiency across varying precipitation levels, allowing it to survive across a broad range of precipitation gradients [68]. In Xinjiang, precipitation is unevenly distributed throughout the year, with most occurring between May and August, accounting for approximately 54.4% of the MAP [69]. Consequently, while MAP reflects the overall precipitation of a site, it does not accurately capture the water availability during a plant’s growth. Given that precipitation is concentrated during the growing season, PWM more effectively represents the moisture availability during this period. We found that the hydraulic traits of *C. mongolicum* were primarily influenced by PWM. Both *D* and *K_th_* increased significantly as PWM decreased. Similar patterns have been observed in other desert shrub species, where hydraulic efficiency may increase under drought conditions as an adaptive response to water-scarce environments [31,70,71]. For instance, a study on Zygophyllaceae desert shrubs in semi-arid and arid regions of northern China reported that vessel diameter and water transport efficiency in roots increased as precipitation decreased [70]. Additionally, in a controlled experiment, *Caroxylon passerinum* (Bunge) Akhani & Roalson exhibited no significant differences in *K_s_* and *K_l_* between drought and control groups post-treatment. However, *K_s_* and *K_l_* were higher in the drought-treated group [71]. *C. mongolicum*, as a C_4_ deciduous plant, exhibits a high capacity for water absorption and storage [72]. High hydraulic conductivity can be adapted to arid environments with high evaporative demand, promoting increased transpiration without increasing the water potential gradient and preventing vessel embolism [73,74]. In addition, species in dry climates have short leaf lifespans and must maximize water transport to fix carbon for dormancy when water is available [10].

Plants in arid environments must adapt flexibly to seasonal fluctuations in moisture to maximize growth advantages with limited resources [75]. Correlation analysis and structural equation modeling revealed that PWM significantly affected the hydraulic traits of *C. mongolicum* and that seasonal precipitation provided an important water source for xylem activity. Liu et al. [29] and He et al. [3] reported that the climate during the growing season has a greater impact on the xylem structure. This is because the activities of the xylem cambium primarily occur during the growing season. Prolonged periods of low or no precipitation can cause cambial activity and temporal periodic changes in cambial activity to cease [76]. A study on *Pinus ponderosa* growing in extremely arid areas indicated that xylem activity is primarily regulated by winter precipitation, independent of temperature [77]. PWM indirectly influences *K_th_* and *VI* by influencing *CA*, which, as an essential mediator, has a significant positive effect on *K_th_* and a significant negative effect on *VI*. *CA* is defined as *VD* multiplied by the mean conduit area [78]; however, similar *CA* values may result in different *K_th_* values depending on the distributional characteristics of the vessels. For example, small vessels within a xylem area form a less efficient xylem than a few large vessels [9]. However, *CA* is positively correlated with *K_th_*, and *CA* increases with the efficiency of water transport. Additionally, *CA* has a significant negative effect on *VI*. Usually, an increase in water-conducting area implies an increase in vessel diameter, and large vessels tend to be more sensitive to xylem embolism [79]. *VI* does not correlate with the mean cavitation pressure, *P_50_*; thus, it is not a good predictor of embolism resistance [15,45]. At the pit level, we found that *C. mongolicum* has a specific vestured pit. Consequently, combining the actual measured *P_50_* and the structure of the striatum will give more accurate results.

## 5. Conclusions

This study demonstrated that *C. mongolicum* adapts to arid environments by optimizing its hydraulic traits and exhibits strong environmental adaptability. The *K_th_* of *C. mongolicum* exhibited the most significant variability, and its higher trait variability improved its adaptability to environmental changes, whereas its safety index *VI* did not change significantly, indicating a conservative trait. The hydraulic traits of *C. mongolicum* exhibited significant coordinate or trade-off relationships, with *K_th_* being significantly positively correlated with *CA* and significantly negatively correlated with (*t/b*)², indicating a trade-off between mechanical support and hydraulic efficiency. *C. mongolicum* enhanced water transport efficiency by augmenting *Kth* and *CA* while regulating the vulnerability to xylem *VD* embolism and ensuring the maintenance of physiological activity when the water supply was low. Additionally, seasonal precipitation prompted *C. mongolicum* to adopt a resource-accessible strategy that enhances water efficiency to ensure rapid access to resources. In conclusion, *C. mongolicum* improves its survival in arid environments by implementing coordinated changes in its hydraulic and anatomical characteristics. This provides an essential theoretical basis for understanding the mechanisms of plant drought adaptation and the selection of sand-fixing shrubs in desert regions.

## Figures and Tables

**Table 1:** Basic characteristics of sample site environment.

Zone	Site	Longitude (°)	Latitude (°)	MAT (℃)	MAP (mm)	AI
Mongolian Autonomous County of Hoboksar	A	86.27	46.18	7.73	144.67	0.089
Urho District	B	85.79	46.17	8.50	147.33	0.088
Mongolian Autonomous County of Hoboksar	C	86.82	45.35	7.91	148.50	0.092
Mongolian Autonomous County of Hoboksar	D	87.07	45.39	7.59	149.00	0.095
Fukang City	E	88.69	44.94	6.86	162.80	0.105
Fukang City	F	88.54	44.95	6.50	175.00	0.116
Fuhai County	G	88.28	45.13	6.14	186.17	0.123

**Table 2:** Summary of variables tested in this study.

Classification	Traits	Abbreviation	Units
Anatomical structure traits	Mean vessel diameter	*D*	μm
	Vessel diameter contributing 95% hydraulic conductivity	*D*_95_	μm
	Hydraulic weighted vessel diameter	*D_h_*	μm
	Vessel density	*VD*	no·mm^−2^
	Percentage of conductive area	*CA*	%
	Vessel grouping index	*V_g_*	
Mechanical strength traits	Inter-wall thickness of the vessel	*t*	μm
	Thickness-to-span ratio of vessels	(*t/b*)^2^	
	Wood density	*WD*	g·cm^−3^
Hydraulic functional traits	Theoretical hydraulic conductivity	*K_th_*	kg·m^−1^·MPa^−1^·s^−1^
	Carlquist’s vulnerability index	*VI*	mm·m^−2^
Climatic variables	Mean annual temperature	MAT	°C
	Mean temperature of driest quarter	TDQ	°C
	Mean temperature of coldest quarter	TCQ	°C
	Mean annual precipitation	MAP	mm
	Precipitation of wettest month	PWM	mm
	Precipitation of driest month	PDM	mm
	Precipitation of wettest quarter	PWQ	mm
	Aridity index (MAP/PET)	AI	

## Data Availability

The data presented in this study are available on request from the corresponding author due to the confidentiality policy of the laboratory.

## Supplementary Materials

The following supporting information can be downloaded at: https://www.mdpi.com/article/10.3390/plants13213005/s1, Figure S1: Pearson correlation analysis of different climatic factors, the lower left corner shows the Pearson correlation coefficient; Table S1: All climate variables downloaded (1970–2000); Table S2: Linear regression analysis of hydraulic traits and climatic factors of *C. mongolicum*. Table S3: The loading of all hydraulic characteristics of *C. mongolicum* on the first two (PC1 and PC2) principal components.
