# Supplementary material for: Intraspecific Variability of Xylem Hydraulic Traits of Calligonum mongolicum Growing in the Desert of Northern Xinjiang, China
Source: Plants (Basel). 2024 Oct 28;13(21):3005. doi: 10.3390/plants13213005 (PMC11548551; doi:10.3390/plants13213005)
Supplement: Supplementary file 1 [file plants-13-03005-s001.zip › Supplementary material.pdf]

Supplementary material

1 Supplementary Tables

Supplementary Table S1. All climate variables downloaded (1970-2000).

Bioclimate variable	Code	Abbreviation
Mean annual temperature	bio1	MAT
Mean diurnal range	bio2	MDR
Isothermality	bio3	Is
Temperature seasonality	bio4	Ts
Max temperature of warmest month	bio5	TWM
Min temperature of coldest month	bio6	TCM
Temperature annual range	bio7	TAR
Mean temperature of wettest quarter	bio8	TWQ
Mean temperature of driest quarter	bio9	TDQ
Mean temperature of warmest quarter	bio10	TWMQ
Mean temperature of coldest quarter	bio11	TCQ
Mean annual precipitation	bio12	MAP
Precipitation of wettest month	bio13	PWM
Precipitation of driest month	bio14	PDM
Precipitation seasonality	bio15	Ps
Precipitation of wettest quarter	bio16	PWQ
Precipitation of driest quarter	bio17	PDQ
Precipitation of warmest quarter	bio18	PWMQ
Precipitation of coldest quarter	bio19	PCQ
Aridity index (MAP/PET)		AI

Supplementary Table S2. Linear regression analysis of hydraulic traits and climatic factors of *C. mongolicum*.

	MAT	TDQ	TCQ	MAP	PWM	PDM	PWQ	AI
D	**0.130***	**0.103***	**0.317*****	0.049	**0.307*****	0.064	**0.123***	0.083
D_{95}	0.093	0.085	**0.349*****	0.038	**0.523*****	**0.127***	**0.253****	0.068
D_h	**0.109***	0.074	**0.246****	0.077	**0.415*****	**0.249****	**0.194****	**0.103***
VD	0.000	0.012	0.026	0.000	0.079	0.036	0.025	0.001
CA	0.045	0.095	**0.279****	0.025	**0.429*****	**0.129***	**0.165***	0.044
V_g	0.026	0.011	0.034	0.007	0.056	0.025	0.047	0.012
t	0.002	0.001	0.000	0.002	0.002	0.001	0.003	0.001
$(t/b)^2$	0.025	0.010	**0.151***	0.005	**0.404*****	**0.122***	**0.280****	0.016
WD	**0.208****	**0.149***	0.089	**0.177****	0.000	0.066	0.038	**0.187****
K_{th}	0.079	0.084	**0.262****	0.063	**0.486*****	**0.277****	**0.213****	0.086
VI	0.027	0.000	0.005	0.012	0.001	0.002	0.002	0.014

Table shows R^2, asterisks represents the significance (* $p < 0.05$, ** $p < 0.01$, *** $p < 0.001$).

Supplementary Table S3. The loading of all hydraulic characteristics of *C. mongolicum* on the first two (PC1 and PC2) principal components.

Hydraulic trait	PC1	PC2
D	-0.335	-0.237
D_{95}	-0.441	-0.208
D_h	-0.442	-0.175
VD	-0.141	0.469
CA	-0.354	0.285
V_g	-0.161	0.095
t	-0.028	-0.433
$(t/b)^2$	0.308	-0.304
WD	0.094	-0.182
K_{th}	-0.088	-0.496
VI	-0.463	0.025
Explained variance proportion	0.372	0.307
Cumulative proportion	0.372	0.679

2 Supplementary Figures

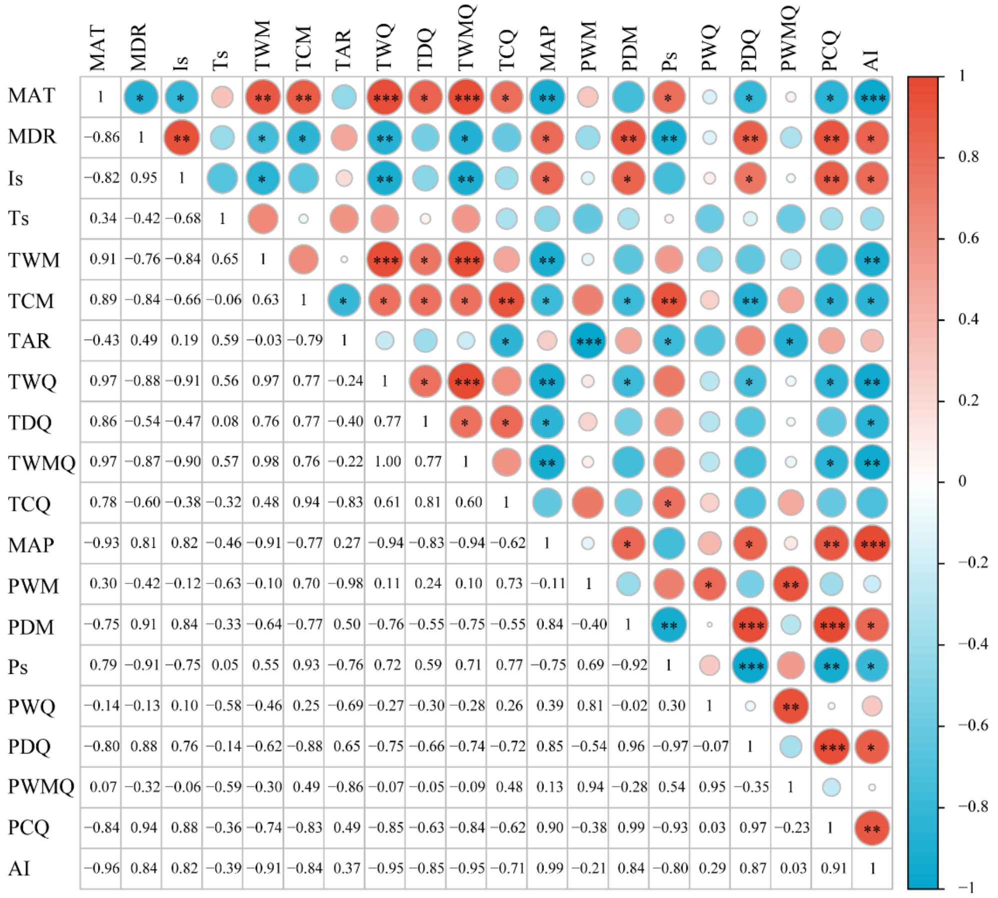

Supplementary Figure S1. Pearson correlation analysis among different climatic factors, the lower left corner shows Pearson correlation coefficient. *P < 0.05; **P < 0.01; ***P < 0.001.
